# Do Clouds Save the Great Barrier Reef? Satellite Imagery Elucidates the Cloud-SST Relationship at the Local Scale

**DOI:** 10.1371/journal.pone.0070400

**Published:** 2013-07-24

**Authors:** Susannah M. Leahy, Michael J. Kingsford, Craig R. Steinberg

**Affiliations:** 1 School of Marine and Tropical Biology, James Cook University, Townsville, Queensland, Australia; 2 Australian Research Council Centre of Excellence for Coral Reef Studies, James Cook University, Townsville, Queensland, Australia; 3 Australian Institute of Marine Science, Townsville, Queensland, Australia; University of Oxford, United Kingdom

## Abstract

Evidence of global climate change and rising sea surface temperatures (SSTs) is now well documented in the scientific literature. With corals already living close to their thermal maxima, increases in SSTs are of great concern for the survival of coral reefs. Cloud feedback processes may have the potential to constrain SSTs, serving to enforce an “ocean thermostat” and promoting the survival of coral reefs. In this study, it was hypothesized that cloud cover can affect summer SSTs in the tropics. Detailed direct and lagged relationships between cloud cover and SST across the central Great Barrier Reef (GBR) shelf were investigated using data from satellite imagery and *in situ* temperature and light loggers during two relatively hot summers (2005 and 2006) and two relatively cool summers (2007 and 2008). Across all study summers and shelf positions, SSTs exhibited distinct drops during periods of high cloud cover, and conversely, SST increases during periods of low cloud cover, with a three-day temporal lag between a change in cloud cover and a subsequent change in SST. Cloud cover alone was responsible for up to 32.1% of the variation in SSTs three days later. The relationship was strongest in both El Niño (2005) and La Niña (2008) study summers and at the inner-shelf position in those summers. SST effects on subsequent cloud cover were weaker and more variable among study summers, with rising SSTs explaining up to 21.6% of the increase in cloud cover three days later. This work quantifies the often observed cloud cooling effect on coral reefs. It highlights the importance of incorporating local-scale processes into bleaching forecasting models, and encourages the use of remote sensing imagery to value-add to coral bleaching field studies and to more accurately predict risks to coral reefs.

## Introduction

The reality of climate change is now well established [1], [2], and there is strong consensus that anthropogenic changes in carbon dioxide, methane, and nitrous oxide are contributing to global warming [2]. In the context of coral reefs, global warming has been implicated in the consistent rise in sea surface temperatures (SSTs) over the past 45 years [3], [4], and consequently to thermal bleaching events [5]. The extent and intensity of the 1998 bleaching event in the Indo-Pacific in particular has drawn attention to the future of coral reefs on a warming planet [5]–[8].

Extensive analyses of bleaching patterns [5], [8], [9], together with comprehensive laboratory experimentation [10]–[12], have dramatically improved our understanding of coral thermal tolerances and our ability to predict future bleaching occurrences [13], [14]. They have also led to the development of a simple and straightforward paradigm regarding critical temperature thresholds: multi-day exposure to SSTs 1–2°C above the long-term local average will cause mass coral bleaching [15]–[17].

While the general mechanisms of anthropogenic global warming are straightforward, feedback loops, particularly involving water vapour, can confound predictions of patterns of warming at the regional and local scales [9], [18]. Atmospheric warming is caused by incident shortwave solar radiation and trapping of the longwave radiation re-emitted by planetary surfaces and “greenhouse gases” [19]. A warm atmosphere promotes evaporation, increasing atmospheric water vapour content while cooling SSTs via the transfer of sensible and latent heat from the ocean’s surface into the atmosphere. At this point, a positive feedback mechanism may be generated, with the “greenhouse” properties of water vapour trapping more heat energy, serving to raise air temperatures and perpetuating a cycle of evaporation and rising air temperatures [20], [21]. Alternatively, a negative feedback mechanism may occur in which increased atmospheric water vapour condenses into clouds; these reflect incident solar radiation, preventing further surface warming [19], [22], [23]. This mechanism has been implicated in distinct events in which coral bleaching thresholds were not attained due to local cloud cover [24]–[30], and has been suggested as a key mechanism constraining tropical SSTs in a warming world [22], [23]. There is merit to both hypotheses, in that observed cloud build-up and temperature responses depend on a number of physical parameters including the strength of convective activity [20], [21] and cloud parameters such as cloud height, altitude, spatial distribution, optical depth, liquid water content, and particle size and state [31]–[33]. These factors determine to what extent local clouds will reflect or transmit incident shortwave radiation, and reflect or transmit outgoing longwave radiation, with measurable consequences for local air and sea temperatures [21], [22], [32], [34], [35].

The complexity of the water vapour feedback mechanisms makes cloud processes one of the major confounding factors in climate models, with a significant proportion of variation between models directly attributable to differences in parametrisation of cloud phenomena [32], [36]. The quality of climate models is further constrained by the scale at which relevant processes are forced, with many models produced on a global, or at best, an ocean basin scale ([13], but see [14], [23]). The output of these models can be downscaled for relevance to ecological management, such as coral reef areas, but the downscaling process is known to reduce the certainty associated with climate predictions, and inaccuracies can be high [37]. Capturing the full range of physical processes involved in climatology, while at the same time producing realistic predictions for local management authorities, is a major challenge in climate modelling; the result is often a disconnect between the predicted regional conditions and the observed local conditions (e.g. [9], [14], [18]).

It is therefore of key importance to collect empirical evidence of atmospheric feedback processes at local (10s of kilometres) to regional (100s to 1,000s of kilometres) scales, and to quantify their effects on incident solar radiation, and subsequently on SST, a direct causative agent of coral bleaching. This study aimed to find empirical evidence of local atmospheric processes, in particular cloud cover, affecting SST on the central Great Barrier Reef (GBR), and to quantify its effect during the vulnerable summer months, when high temperatures and generally low convective activity increase the probability of mass thermal bleaching events. Records of SST, incident solar radiation, and cloud cover during the multiyear period from 2004 to 2008 were retrieved from a combination of *in situ* loggers and satellite imagery and were successfully used to identify relationships between the variables, including responses that were temporally lagged, at different positions across the GBR shelf.

## Materials and Methods

### Study Period

The Austral summer on the GBR extends from October to March, with incident solar radiation peaking around December and SSTs peaking between December and February [38]. The atmospheric circulation of the Australian summer monsoon increases cloud cover and brings in weaker and moister surface winds during this time [38], such that cloud cover and rainfall are highest in February [39]. Thermal bleaching risk is highest during this period, with particularly dramatic mass bleaching events recorded in the Austral summers of 1998 [5] and 2002 [9].

### Study Area

**Figure 1:**
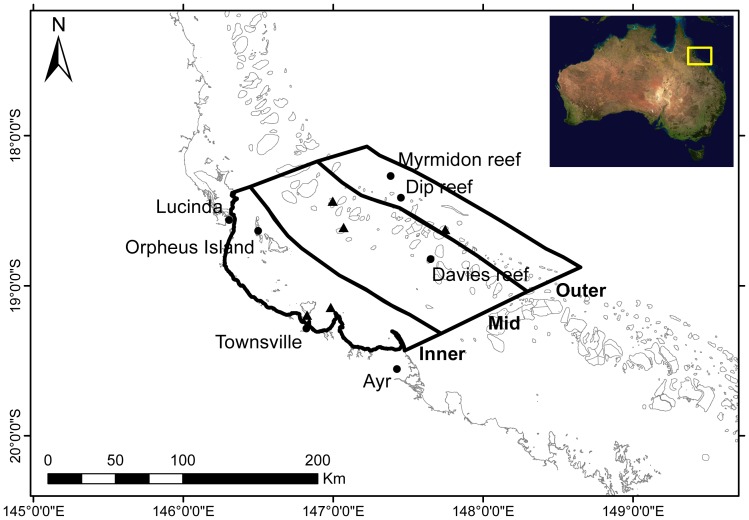
Study area. Location and extent of cross-shelf study regions and AIMS monitoring loggers used in this study. Named reefs indicate loggers used in this study. Black-fill triangles indicate loggers used to validate the generality of SST trends for each study region. Inset: approximate location of the study area; composite satellite image of Australia courtesy of NASA (2002) MODIS technology.

It was hypothesized that cloud cooling effects would vary with distance from shore, potentially due to orographic effects [40], as well as a coastal-to-ocean gradient in both bathymetry and exposure, in which features an inner shelf open coastal lagoon, a mid shelf complex reef matrix, and an outer shelf exposed to the Coral Sea [41], [42]. Environmental data was therefore collected from Australian Institute of Marine Science (AIMS) island and buoy weather stations deployed at sites at inner (Orpheus Island, 18°36′46.08″S, 146°28′59.16″E; Cleveland Bay, 19°8′27.6″S, 146°53′23.4″E; and Middle Reef, 19°11′40.2″S, 146°48′36.72″E), mid (Davies Reef, 18°49′53.82″S, 147°38′4.2″E; John Brewer Reef, 18°37′15.24″S, 147°3′13.68″E; and Kelso Reef, 18°26′42.84″S 146°59′32.06″E), and outer shelf (Dip Reef, 18°24′5.33″S, 147°27′3.67″E; Chicken Reef, 18°39′17.57″S, 147°43′15.49″E; and Myrmidon Reef, 18°16′27.29″S, 147°22′54.25″E) positions (Fig. 1). These distance strata were also of biological interest, as substantial variation in marine assemblages are found cross-shelf (e.g. soft corals [43], sponges [44], hard corals [45], herbivorous fishes [46]).

“Regions of interest” that equated to major cross-shelf positions on the central GBR were produced in ESRI ArcGIS 10.0, using shapefiles of GBR features and management regions defined and provided by the Great Barrier Reef Marine Park Authority (GBRMPA) as UTM Zone 55 projections, GDA94. Management regions were modified using expert opinion to produce inner (6,734 km^2^), mid (10,417 km^2^), and outer shelf (5,471 km^2^) study regions, extending along the coastline from Hinchinbrook Island (18°21′0.00″S, 146°17′49.20″E) south to Cape Bowling Green (19°24′46.80″S, 147°28′8.40″E) and covering the full cross-shelf area (Fig. 1).

### Collection and Processing of SST Data

Multi-year SST data was available from multiple near-surface temperature loggers in each shelf position (inner: Orpheus Island, Cleveland Bay, Middle Reef; mid: Davies Reef, John Brewer Reef; outer: Dip Reef, Chicken Reef). As SST trends were consistent within each shelf position, further analyses were conducted using data from loggers with the longest uninterrupted reports between 1 October 2004 and 31 March 2008 at each shelf position (inner: Orpheus Island, mid: Davies Reef, outer: Dip Reef). Multi-year comparisons were produced from expert quality-controlled data from loggers at similar and biologically relevant depths (3–6 m). Thermal readings were taken every 30 minutes. Datasets were trimmed to include only peak heating hours (1100–1600 h, comparable to [40]), and were averaged by day, producing a “mean daily daytime SST.” Within-day variation was low, and was generally attributable to tidal fluxes. Preliminary analyses indicated that two summers in the study period were particularly warm (2005 and 2006), while two were relatively cooler (2007 and 2008). These are distinguished graphically for all relevant analyses.

The full SST datasets were detrended using a 21 day moving average in order to remove the seasonal component of variation in the data. Further analyses were conducted on the residuals or “white noise” in the dataset as per Chatfield [47].

### Collection and Processing of Cloud Cover Data

Pre-processed remotely-sensed cloud cover information was used to address the question of cloud cooling effects on the central GBR. Cloud imagery was collected using the Moderate Resolution Imaging Spectroradiometer (MODIS) mounted on board the Terra and Aqua satellites. Images were pre-processed to Level 2 Cloud Product state using MOD06 cloud retrieval algorithms as described in King et al. [48]. Images were downloaded from NASA’s LAADS server (ladsweb.nascom.nasa.gov, collection 5.1) at a resolution of 5×5 km and projected as a Universal Transverse Mercator (UTM) Zone 55, WGS84.

For each image, the cloud fraction layer was extracted from the original Level 2 Cloud Product file and reclassified from a 0–255 RGB range to a binomial “yes/no” cloud present in each 5×5 km pixel. The reclassified layer was then trimmed to the regions of interest defined above, and the number of yes-cloud and no-cloud pixels in each study region was recorded and converted to a percent cloud cover. Information from satellite imagery that provided only partial coverage of the study region was discarded if it contained <50% coverage of each shelf position, i.e. <130.5, 208.5, or 110 informative pixels for the inner, mid, or outer shelf study areas, respectively. Only daytime imagery was used for analyses presented here, i.e. one Terra pass at ∼1200–1300 h and one Aqua pass at ∼1500–1600 h.

### Collection and Processing of Radiation Data

Records of incident Photosynthetically Active Radiation (PAR) were collected to serve as proxies for the amount of light reaching the ocean’s surface. Measurements of PAR indicate incident visible light in the 400–700 nm range, which is only a fraction of total incident solar radiation (“insolation,” ∼100–14,000 nm) and is monitored for its relevance to primary productivity [49]. The ratio of PAR to insolation is approximately constant across a range of cloud conditions [31], [50] and so is considered a reliable proxy for insolation.

Measurements of PAR, integrated over 10 minutes, were recorded in µmol·s^−1^·m^−2^ every 30 minutes between 1 October 2004 and 1 March 2008 from AIMS monitoring stations at all three shelf positions (inner: Orpheus Island; mid: Davies Reef; outer: Myrmidon Reef). PAR datasets were trimmed to include only peak heating hours (1100–1600 h) and were then averaged by day, producing a “mean daily daytime PAR.”

### Data Analysis

Daytime daily average SST, cloud cover, and PAR during the peak bleaching period (January to March of each study year) were analysed using a 3-way fixed factor ANOVA to test for the effects of study summer (2005 to 2008), month (January to March), shelf position (inner, mid, or outer shelf), and their interactions. The daily average value of each variable at each shelf position was treated as an independent replicate. The cloud cover dataset was arcsine transformed (asin(√value)*180/π) to meet the assumptions of ANOVA [51]. The SST and PAR datasets violated the assumption of normality despite transformations; therefore a more conservative critical *p* value (*p*<0.01) was applied to these data [52].

To test the hypothesis that cloud cover intercepts incident solar radiation, the smoothed time series (five-day moving averages) of PAR was regressed against smoothed arcsine transformed cloud cover for each study summer in each study region.

Overlayed smoothed time series (five-day moving average) of SST and cloud cover were used for qualitative and quantitative assessments of the relationship between the two variables and identification of potential lags between cloud phenomena (increase or decrease in cloud cover) and SST responses. The pattern and strength of the relationship between arcsine transformed daily cloud cover and detrended daily SST (i.e. SST residuals) was then quantified for ±10 time lags using a cross-correlation (SYSTAT 12.02.00). The lags producing the strongest relationship between the two variables were incorporated into a linear regression for each shelf position in each study summer. When detrended SST was lagged *after* the cloud cover dataset, it was considered to be the dependent variable and was regressed against transformed cloud cover. When detrended SST was lagged *before* the cloud cover dataset, cloud cover was considered the dependent variable and was regressed against detrended SST. Directionality of all regressed relationships is indicated in-text and in relevant tables.

## Results

### Spatial and Temporal Variation in SST

**Figure 2:**
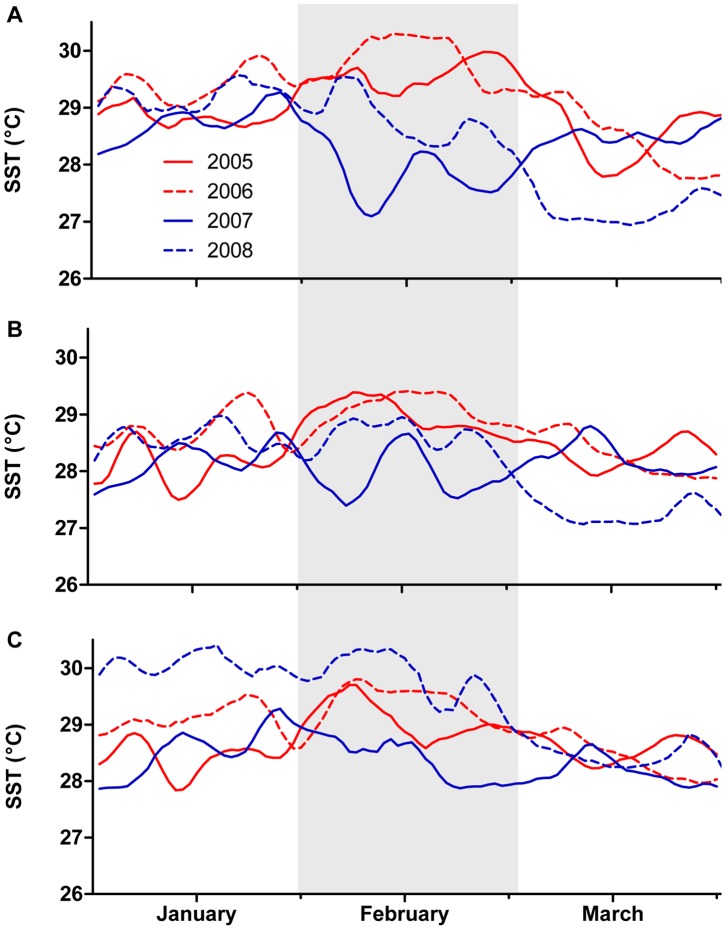
SST time series. Summer daytime SSTs in the (A) inner, (B) mid, and (C) outer shelf study regions. SSTs are smoothed using five-day moving averages. Red lines denote summers with warmer thermal profiles (2005 and 2006); blue lines denote summers with cooler thermal profiles (2007 and 2008). The period of greatest difference in SST, cloud cover, and PAR between the warmer and cooler summers (discussed in-text) is highlighted in grey.

There was great temporal variation in SST at the inner and mid shelf positions both within and among years, serving to identify two years with generally warmer summers (2005 and 2006) and two years with generally cooler summers (2007 and 2008, Fig. 2A, B). Summer SSTs also varied significantly by shelf position. Variation by shelf position by summer and month resulted in a significant three-way interaction (Table 1). The pattern of SST buildup, peak, and decline between January and March of each summer demonstrated a clear seasonal trend, with SST maxima generally attained in February of each study summer (Fig. 2A, B). In contrast, in the outer shelf region, there was one cool (2007) and three warm summers (2005, 2006, and 2008), and there was a less distinct temperature peak in February (Fig. 2). Summer 2007 was consistently the coolest across all shelf positions.

**Table 1:** ANOVAs that tested sea surface temperature (SST), photosynthetically active radiation (PAR), and arcsine transformed cloud cover by shelf position, summer, month, and their interactive effects.

	Factor	d.f.	MS	F	p
SST	Shelf position	2	26.194	181.17	**<0.001**
	Summer	3	25.323	175.146	**<0.001**
	Month	2	55.389	383.097	**<0.001**
	Shelf position * Summer	6	10.959	75.798	**<0.001**
	Shelf position * month	4	1.949	13.482	**<0.001**
	Summer * month	6	20.06	138.744	**<0.001**
	Shelf position * summer * month	12	0.817	5.652	**<0.001**
	Error	1,047	0.145		
Cloud cover	Shelf position	2	1,553.80	2.395	0.092
	Summer	3	15,817.78	24.382	**<0.001**
	Month	2	4,551.47	7.016	**0.001**
	Shelf position * Summer	6	49.439	0.076	0.998
	Shelf position * month	4	109.143	0.168	0.955
	Summer * month	6	10,106.87	15.579	**<0.001**
	Shelf position * summer * month	12	123.409	0.19	0.999
	Error	1,047	648.748		
PAR	Shelf position	2	35,126,017.63	167.626	**<0.001**
	Summer	3	23,773,571.26	113.451	**<0.001**
	Month	2	4,387,579.55	20.938	**<0.001**
	Shelf position * Summer	6	575,370.67	2.746	0.012
	Shelf position * month	4	3,479,080.00	16.603	**<0.001**
	Summer * month	6	8,987,451.70	42.889	**<0.001**
	Shelf position * summer * month	12	879,638.44	4.198	**<0.001**
	Error	1,047	209,549.94		

SST and PAR violated the assumption of normality and are therefore interpreted with a more conservative *p*<0.01.

### Spatial and Temporal Variation in Cloud Cover

**Figure 3:**
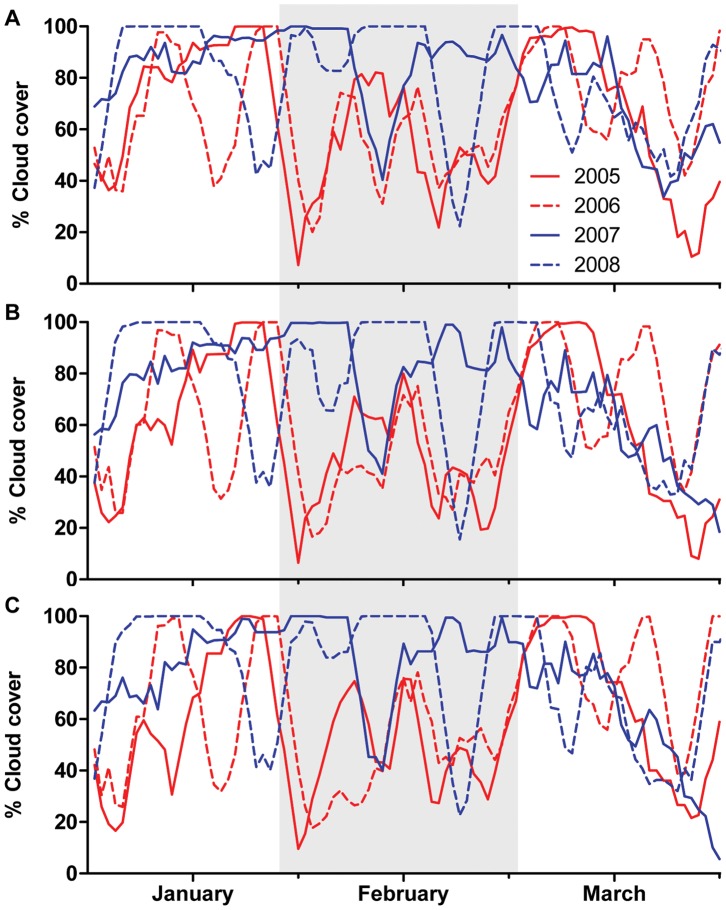
Cloud time series. Summer daytime cloud cover in the (A) inner, (B) mid, and (C) outer shelf study regions. Cloud cover is smoothed using five-day moving averages. Red lines denote summers with warmer thermal profiles (2005 and 2006); blue lines denote summers with cooler thermal profiles (2007 and 2008). The period of greatest difference in SST, cloud cover, and PAR between the warmer and cooler summers (discussed in-text) is highlighted in grey. The spike in cloud cover in late March 2006 is due to the path of Tropical Cyclone Larry.

**Figure 4:**
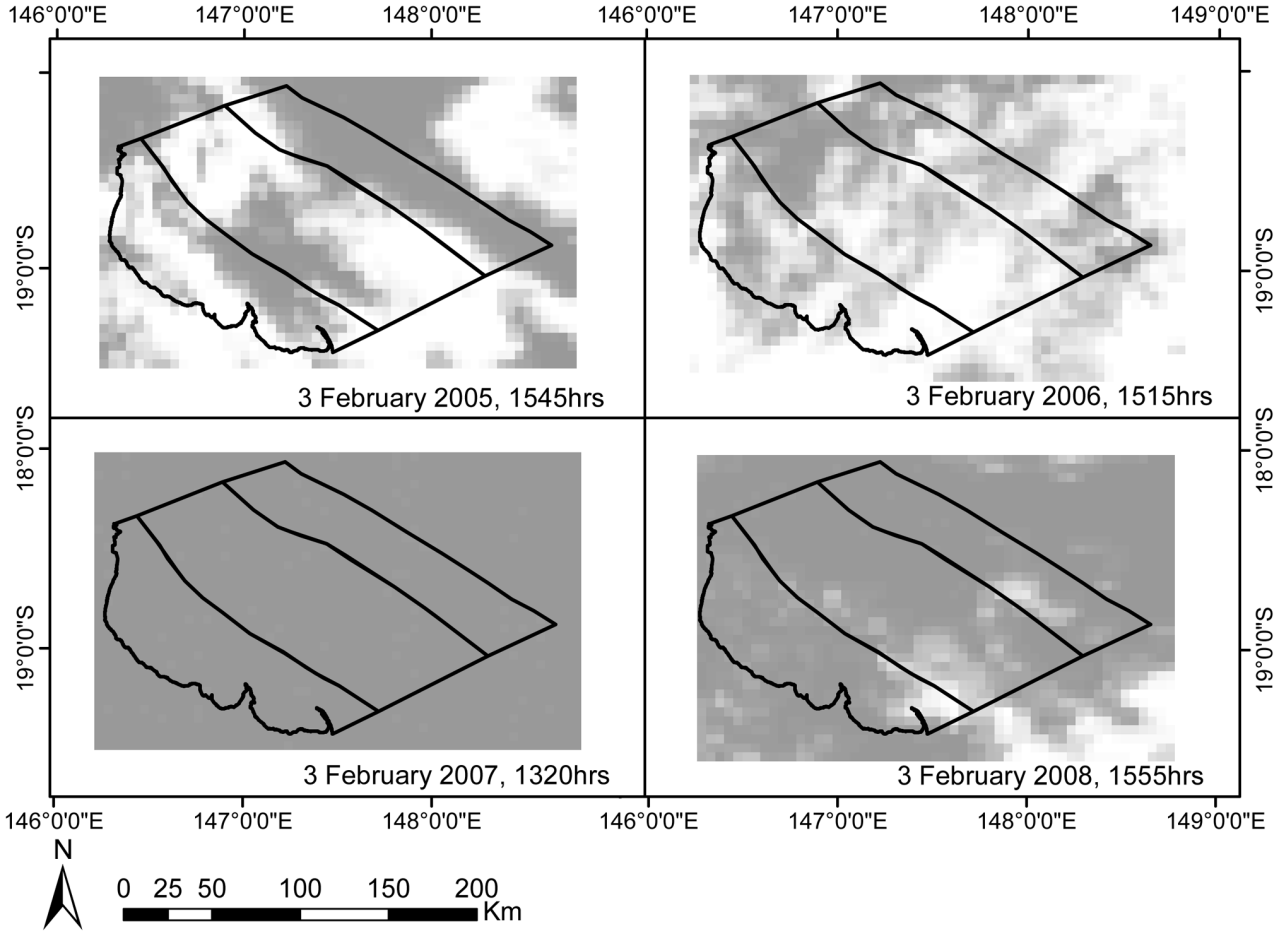
Sample MODIS imagery. Images were taken on the same day in each of the four study summers. Images have been reclassified to Y/N cloud present (grey: yes cloud; white: no cloud). Black polygons indicate study regions (inner, mid, and outer shelf). Cloud cover illustrated in each image is approximately representative of mean and median cloud cover for February of each study summer.

Cloud cover did not vary significantly by shelf position, but did vary significantly by summer and by month, and by summer*month interaction (Fig. 3, Table 1). Cloud cover was consistently high in January, February, and early March of 2007 and 2008, and declined in mid-March of both 2007 and 2008 (Fig. 3A–C). The trend was different in 2005 and 2006, with high cloud observed across all shelf positions in January and early March, but not in February (Fig. 3A–C). The nature of cloud cover also differed between study summers, with typically patchier cloud cover noted in 2005 and 2006, and consistently higher and more extensive cloud cover observed in 2007 and 2008 (Fig. 4).

### Spatial and Temporal Variation in Incident Radiation

**Figure 5:**
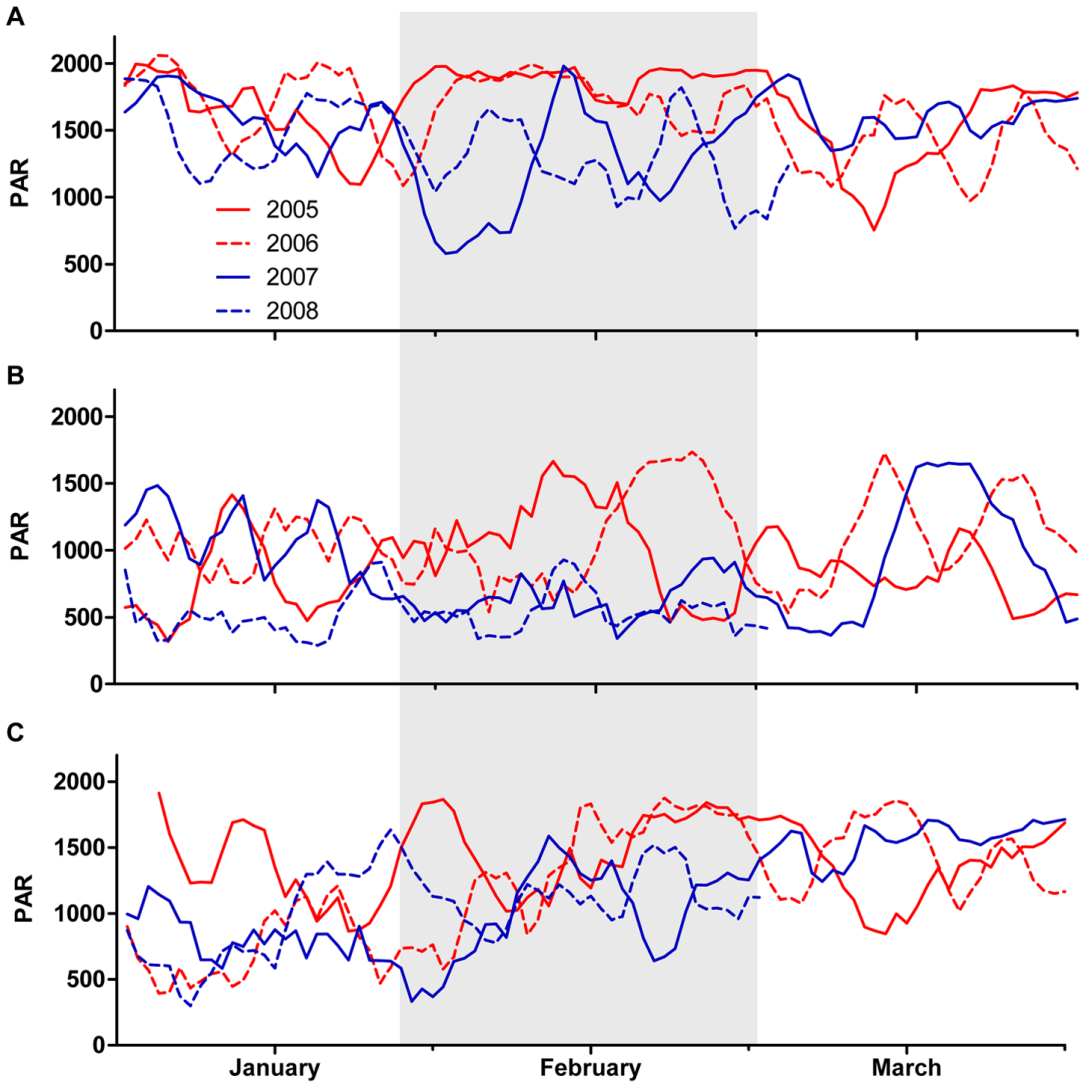
PAR time series. Summer daytime PAR (Photosynthetically Active Radiation, in µmol·s^−1^·m^−2^) in the (A) inner, (B) mid, and (C) outer shelf study regions. PAR is smoothed using five-day moving averages. Red lines denote summers with warmer thermal profiles (2005 and 2006); blue lines denote summers with cooler thermal profiles (2007 and 2008). The period of greatest difference in SST, cloud cover, and PAR between the warmer and cooler summers (discussed in-text) is highlighted in grey.

PAR varied significantly among shelf positions, study summers, and months, resulting in a significant three-way interaction (Table 1). However, clear patterns were found among summers at all shelf positions, with the greatest differences among summers occurring in late January and early to mid February (Fig. 5). In the inner shelf region, mean daytime PAR was high across all four study summers, with the exception of a large (500–1,000 µmol·s^−1^·m^−2^) drop in late January 2007 and 2008, which persisted throughout the month of February (Fig. 5A). The same relative difference in PAR between warm (2005 and 2006) and cool (2007 and 2008) years was observed in the mid shelf region, but details of the pattern differed. In the mid shelf region, consistently low summer PAR values gave way to a distinct rise (of approximately 1,100 µmol·s^−1^·m^−2^) in early February 2005 and late February 2006, which persisted for approximately two weeks (Fig. 5B). A similar, albeit weaker, pattern occurred in the outer shelf region, where PAR values rose approximately 700 µmol·s^−1^·m^−2^ higher in late February 2005 and 2006, as compared to the same period in 2007 and 2008 (Fig. 5C).

Incident solar radiation in the PAR range was generally a strong proxy for cloud cover, although the strength of the relationship varied in both time and space (Table 2). The relationship between cloud cover and PAR was consistently strong and negative across all shelf positions in almost all study summers; cloud cover accounted for up to 55.76% of the variation in PAR (summer 2006, inner shelf, Table 2). Among summers, the fraction of PAR explained by cloud cover was generally highest in the inner shelf region, was lowest mid shelf, and was intermediate in the outer shelf. There is no clear pattern between summers (Table 2).

**Table 2:** Regressions between 5-day moving averages of PAR and arcsine transformed cloud cover across all shelf positions in all study summers.

Shelf position		Summer 2005	Summer 2006	Summer 2007	Summer 2008
**Inner**	**R^2^**	0.471	0.558	0.343	0.465
	**p**	**<0.001**	**<0.001**	**<0.001**	**<0.001**
	**N**	90	90	90	63
**Mid**	**R^2^**	0.026	0.125	0.088	0.085
	**p**	n.s.	**<0.001**	**0.005**	**0.023**
	**N**	90	90	90	61
**Outer**	**R^2^**	0.378	0.007	0.468	0.231
	**p**	**<0.001**	n.s.	**<0.001**	**<0.001**
	**N**	87	90	90	61

All significant relationships were negative.

### Relationship between Cloud Cover and SST

**Figure 6:**
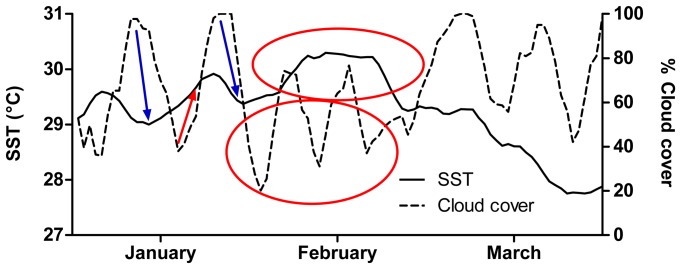
Sample cloud cover and SST overlay. Visualization of lags between cloud cover phenomena and SST responses; summer 2006, inner shelf position. Blue arrows indicate suggested causative lagged cloud cooling events; red arrows and circles indicate suggested causative lagged warming events.

**Figure 7:**
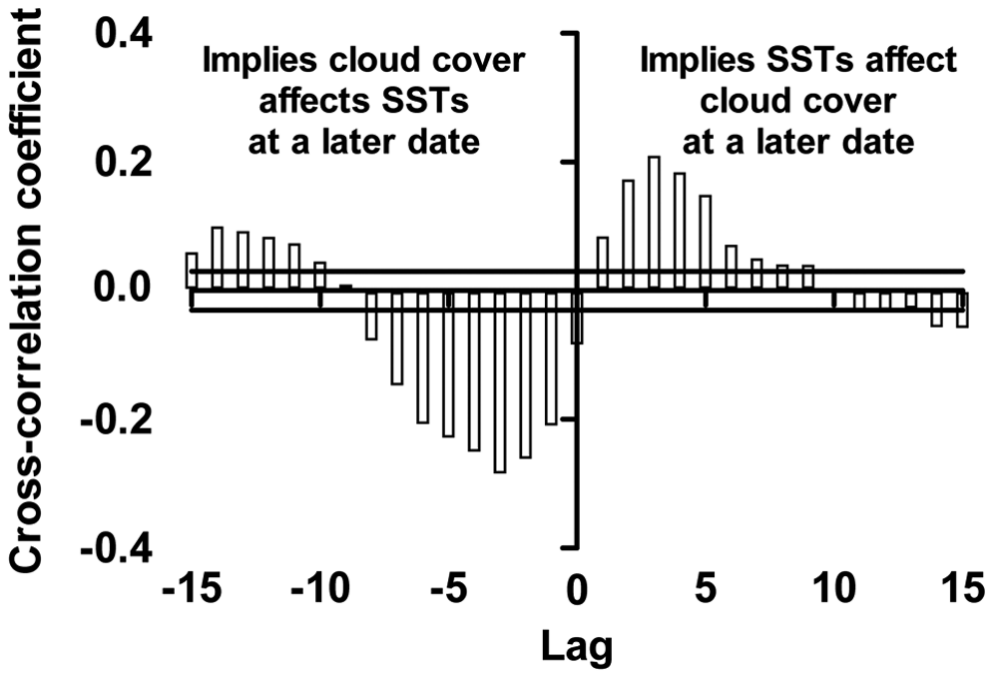
Cross-correlation output. Cross-correlation of cloud cover against SSTs, across all study regions and summers. Note the negative peak in the relationship at −3 days of lag, and the positive peak in the relationship at +3 days lag.

A direct comparison of cloud cover and SST time series indicated a lagged relationship between the two variables, with distinct cloud events (increases or decreases) followed by inverse SST changes (decrease or increase) several days later (Fig. 6). For example, two relatively short “pulses” of cloud in early January 2006 were associated with subsequent declines in SST of approximately 0.5°C, while oscillating low and medium cloud cover in early February was associated with high SST in mid to late February (Fig. 6). Formal analysis of the lagged relationship using a cross-correlation indicated two major peaks in the correlation between cloud cover (arcsine transformed) and SST (detrended; residuals only, Fig. 7). The first peak indicated a strong negative correlation between cloud cover and SST residuals three days later (lag: −3). The second peak indicated a strong positive correlation between cloud cover and SST residuals three days prior (lag: +3), which can instead be expressed as a positive correlation between SST residuals and cloud cover three days later (Fig. 7).

Regressing cloud cover (arcsine transformed) against SST residuals lagged three days later produced significant relationships, with cloud cover explaining between 2.1 and 32.1% of the variation in SST residuals three days later (Table 3). This was true over most of the study region in almost all study summers (Table 3). The relationship was significant in 2005 at all shelf positions, explaining between 15.4 and 32.1% of the variation in lagged SST. Cloud cover significantly affected SST residuals across all shelf positions in 2008, but to a lesser extent than in 2005, explaining only 6.6 to 13.1% of the variation (Table 3). The relationship between cloud cover and lagged SST residuals was fairly weak in both 2006 and 2007. The regression was significant in the mid shelf region in all study summers, explaining between 5.5 and 17.7% of the variation. The strongest regression occurred in the inner shelf region in 2005 (32.1%, table 3).

**Table 3:** Transformed cloud cover regressed against SST residuals three days later, across all shelf positions and study summers.

Shelf position		Summer 2005	Summer 2006	Summer 2007	Summer 2008
**Inner**	**R^2^**	0.321	0.020	0.012	0.131
	**p**	**<0.001**	n.s.	n.s.	**<0.001**
	**N**	87	87	87	88
**Mid**	**R^2^**	0.177	0.081	0.055	0.066
	**p**	**<0.001**	**0.008**	**0.030**	**0.015**
	**N**	87	87	87	88
**Outer**	**R^2^**	0.154	0.103	0.006	0.100
	**p**	**<0.001**	**0.002**	n.s.	**0.003**
	**N**	87	86	87	88
**Overall**	**R^2^**	0.208	0.061	0.021	0.096
	**p**	**<0.001**	**<0.001**	**0.020**	**<0.001**
	**N**	261	260	261	264

All significant relationships were negative, i.e. increased cloud cover was associated with subsequent decreases in SST.

Regressions of SST residuals against arcsine transformed cloud cover lagged three days later (i.e. SST affecting cloud cover) identified a much weaker overall relationship, with fewer significant relationships within each study summer, and a totally non-significant relationship in 2005 (Table 4). The relationship was strongest in 2008, with SST residuals explaining between 9.1 and 21.6% of the variation in cloud cover three days later. The overall significance of the relationship in 2006 and 2007 was driven by individual shelf positions in each study summer (9.9% in the mid shelf in 2006, 12.6% in the inner shelf in 2007, Table 4). The regressions were most frequently significant in the inner shelf region, and appear to show a cross-shelf gradient in 2008, with SST residuals explaining the highest variation in lagged cloud cover in the outer shelf region (21.6%).

**Table 4:** SST residuals regressed against transformed cloud cover three days later, across all shelf positions and study summers.

Shelf position		Summer 2005	Summer 2006	Summer 2007	Summer 2008
**Inner**	**R^2^**	0.001	0.044	0.126	0.091
	**p**	n.s.	**0.051**	**<0.001**	**0.004**
	**N**	87	87	87	88
**Mid**	**R^2^**	0.003	0.099	0.037	0.174
	**p**	n.s.	**0.003**	0.073	**<0.001**
	**N**	87	87	87	88
**Outer**	**R^2^**	0.016	0.040	0.018	0.216
	**p**	n.s.	0.075	n.s.	**<0.001**
	**N**	87	86	87	88
**Overall**	**R^2^**	0.004	0.058	0.049	0.152
	**p**	n.s.	**<0.001**	**<0.001**	**<0.001**
	**N**	261	260	261	264

All significant relationships were positive, i.e. rising SSTs were associated with subsequent increases in cloud cover.

## Discussion

### Cloud Cooling Effects on Lagged SST

The combination of *in situ* SST data and remotely-sensed cloud imagery used in this study indicated a significant, albeit variable, relationship between cloud cover and lagged SST, with cloud cover explaining up to 32.1% of the variation in SST three days later (Table 3). Our data suggest that the SST response to cloud cover is primarily a result of cloud interception of solar radiation (Table 2). This work provides the first empirical evidence of a phenomenon that has until now been reported only anecdotally in the literature on coral bleaching [9], [24]–[30], [53]. While the capacity for specific cloud types to limit or reduce SSTs is well known, particularly in the physical modelling literature [2], [21], [22], [54], [55], to our knowledge, this study provides the first quantitative assessment of cloud cooling effects in coral reef systems.

While this study focused on the effects of cloud cover alone, other cloud parameters (e.g. cloud height, altitude, optical depth, liquid water content, and particle size and state [31], [32]) and other environmental variables (e.g. evaporation from wind and lagoonal effects such as tidal mixing, wind mixing, and local currents [56] [41]) are also known to affect SSTs at spatial scales of kilometres to hundreds of kilometres. These factors can alter patterns in the interception of solar radiation, the mixing of water masses, or air-sea heat exchange processes. Furthermore, some unexplained variation in our study may be a result of the insensitivity of the regressions to short “pulse” cloud events that may have a cumulative effect on SST (e.g. Fig. 6). The addition of these factors to our analysis could explain a greater proportion of the variation in SST and lagged SST.

However, given the number of variables involved in thermal forcing on coral reefs [41], [42], the extent to which cloud cover alone explained variation in lagged SST is surprisingly large. Furthermore, the peak in the strength of the relationship with a three day lag was not particularly distinct, suggesting a great deal of inertia in the system, with cloud build-up inducing a slow, gradual, and persistent SST response. Indeed, the cross-correlation indicated a weaker, but still significant relationship between cloud cover and subsequent SST responses with lags anywhere between 0 and 8 days, which indicated both a rapid SST response to changes in cloud cover (0 day lag), and a persistent effect (up to 8 day lag, Fig. 7).

The greatest variation in the strength of the cloud-SST relationship was among study summers. However, the strength of the regression was not associated with summers with similar thermal profiles (i.e. “warmer” 2005 and 2006 versus “cooler” 2007 and 2008), as was originally expected. Instead, cloud cooling effects were found to be consistently strongest in 2005 and 2008, which exhibited strongly differing thermal profiles (warmer and cooler, respectively). The El Niño Southern Oscillation (ENSO) strongly influences regional atmospheric circulation during the austral summer [57] and is frequently associated with sustained SST anomalies on the GBR [39], [58]. The ENSO phenomenon may influence the variation in the strength of the cloud cover-SST relationship identified in this study, as the strongest cloud cooling effects were noted in strongly ENSO summers: 2005 experienced a strong El Niño summer, and 2008 a strong La Niña summer. The other study summers (2006 and 2007) experienced neutral Southern Oscillation Indices [59]. Our results indicated that the extent to which cloud cover was responsible for cooling SST was strongest in a hot, dry El Niño summer (2005), but was also important during an overcast, cool La Niña summer (2008, Table 3). The importance of the cloud cooling effect in the El Niño summer may be due to the reduced activity of other cooling mechanisms such as surface winds [39], [57] and higher initial SSTs (both from local heating and from advection of warm water via the South Equatorial Current [42]). The strong cloud cooling effect observed in the La Niña summer may in part be due to the extent of cloud cover in that study summer (Fig. 3 and 4), as well as the effect of cloud cover covariates such as wind speed [60].

The spatial scale at which the study was carried out did not indicate consistent cross-shelf patterns; however, cloud cover explained the greatest proportion of SST variation in the inner shelf in both 2005 and 2008, which were the strongly ENSO summers in which the overall cloud cooling effect was greatest. This may be a result of local orographic effects [40], in which clouds “pile up” against the Great Dividing Range, a coastal mountain range in this region. This explanation is supported by the strength of the observed relationship between cloud cover and PAR, which was generally highest in the inner shelf region, indicating the presence of optically thicker clouds in this area. Reduced transmittance of solar radiation in the PAR range is indicative of low-altitude clouds [50], which have high albedo, and therefore strong cooling effects, due to characteristics such as small-radius, high particle density, liquid-phase water droplets [33]. Greater radiation reflection or interception by clouds in the inner shelf region may be particularly beneficial to coral reefs in this area, where bleaching patterns are best described by radiation patterns rather than by SSTs [61].

Cloud cooling effects were weaker in the mid shelf region than in the inner shelf region, but were significant across all four study summers (Table 3), suggesting that cloud cover represents a natural means of regulating SST in this region, where water column mixing is limited by the reduced tidal currents characteristic of the dense reef matrix [41].

### Rising SSTs and Subsequent Cloud Buildup

Changes in SST explained up to 21.6% of the variation in cloud cover three days later (Table 4). Taking into account subsequent cloud cooling effects on SST, this is indicative of a negative cloud feedback mechanism in this system. The strong positive relationship between SST and cloud cover was only consistently observed in 2008, the La Niña study summer. This may be a consequence of the lower barometric pressure and greater wind strength characteristic of La Niña summers in this part of the world [39] increasing local evaporative activity.

A cross-shelf gradient in the strength of the SST-cloud relationship was only observed in 2008, with SST residuals explaining the greatest proportion of the variation in cloud cover three days later in the outer shelf region. This may be a result of a greater evaporation rate offshore, where warm, highly saline water “puddles” [41], forming clouds which are then pushed inshore, where their subsequent cooling effect is strongest.

The increase in cloud cover following the rises in SST reported here (Table 4) can be attributed to increased evaporative activity, as is likely the case in the inner shelf region [41]. However, other potential influences may be operating in this system, including biological feedback mechanisms. For example, observed cloud build-up may be a response to local increases in the aerosol particles serving as Cloud Condensation Nuclei. The waters of the GBR are known to be a significant source of these aerosols [62], which are primarily composed of dimethylsulfide (DMS) [63], [64]. The DMS precursor, dimethylsulphoniopropinate (DMSP), is produced by marine phytoplankton [33] and coral-symbiont dinoflagellates [65]–[67]. Atmospheric DMS production by corals increases with increased light intensity and SST, but declines if corals are exposed to prolonged thermal and light stress [68]. On a global scale, DMS is involved in a negative feedback loop with incident solar radiation and cloud cover [69]. Part of the SST-cloud relationship observed here may therefore be a result of DMS production by corals on the central GBR, particularly in the coral-rich mid shelf region [68], [70]. This coral-produced atmospheric DMS may increase local densities of cloud condensation nuclei enough to promote the formation of high albedo clouds (composed of high-density small-radius liquid-phase droplets [33]).

### Implications

We have demonstrated that cloud cover is an important determinant of SST, which has direct implications for thermal coral bleaching. From a computational or modelling perspective, our results highlight the importance of incorporating local sources of variation in order to better align model predictions with the reality of coral bleaching patterns on a local scale (10s to 100s of kilometres). The current push towards incorporating local physical processes and increasing the spatial resolution of models is producing dramatic improvements in model predictive abilities [14], [71]. We recommend the inclusion of quantitative cloud parameters (e.g. cloud cover, but also optical depth) in order to maximize model relevance for reef managers and coral reef biologists.

From a biological perspective, our work underscores the importance of quantifying local environmental conditions within the context of basin-scale physical processes (e.g. ENSO) when assessing imminent, current, or historical bleaching records. Thermal bleaching reports frequently note the occurrence of “doldrum” periods prior to and during a mass bleaching event [5], [26], [30]. Despite the fact that the direct (decreased UV stress [15], [34]) and indirect (decreased SST [this paper, 9,24]) benefits of cloud cover are well-recognized, the information available from satellite imagery remains a largely underutilised resource. The use of remote sensing technologies such as satellite imagery can be used for both atmospheric forecasting and hindcasting, substantiating observations made in the field, value-adding to bleaching reports, and improving our predictive abilities and *a posteriori* understanding of bleaching events.
